# Impressive long-term response with chemo-endocrine therapy in a premenopausal patient with metastatic breast cancer: A case report

**DOI:** 10.1097/MD.0000000000020396

**Published:** 2020-06-12

**Authors:** Roberta Maltoni, Michela Palleschi, Giulia Gallerani, Sara Bravaccini, Lorenzo Cecconetto, Elisabetta Melegari, Mattia Altini, Andrea Rocca

**Affiliations:** Istituto Scientifico Romagnolo per lo Studio e la Cura dei Tumori (IRST) IRCCS, Meldola, Italy.

**Keywords:** bone lesion, endocrine therapy, metastatic breast cancer, partial remission

## Abstract

**Rationale:**

Patients with, or who develop, metastatic breast cancer have a 5-year relative survival of about 25%. Endocrine therapy clearly improves outcomes in patients with estrogen receptor-positive breast cancer. In the metastatic setting, the primary goal of treatment is to maintain long-term disease control with good quality of life. Rarely, exceptional responders achieve durable disease control, and potential cures cannot be ruled out.

**Patient Concerns:**

We report the case of a 39-year-old woman with primary breast cancer and associated synchronous bone metastases, who experienced a disease response of 12 years with hormonal therapy as maintenance after first line chemotherapy, with a good toxicity profile.

**Diagnosis:**

The patient was diagnosed with estrogen receptor + human epidermal growth factor receptor 2 (HER2)− metastatic breast cancer with synchronous bone metastases.

**Interventions:**

This patient was treated with chemotherapy for 6 cycles as a first-line therapy following by endocrine treatment given as a maintenance therapy.

**Outcomes:**

Our patient experienced a progression-free survival >12 years with an exceptionally good quality of life.

**Lessons:**

Our anecdotal experience highlights the existence of exceptional responders among patients with hormone receptor-positive metastatic breast cancer, who achieve clinical remission and durable disease control with endocrine therapy. Being able to identify these patients could help in the selection of the best treatment option among the many available.

## 1 Introduction

Breast cancer is the most prevalent cancer in women, in the developed world. Long-term survival outcomes are related to disease stage at presentation. Patients with, or who develop, metastatic breast cancer have a 5-year relative survival of only about 25%,^[1]^ with somewhat better outcomes in de novo metastatic disease than in recurrent metastatic breast cancer, particularly after a short disease-free interval.^[2–4]^ The most common subset in both the early- and late-stage setting is hormone receptor (HR)-positive breast cancer, with more than 70% of tumors expressing HRs.^[5]^ In patients with estrogen receptor (ER)-positive breast cancer, endocrine therapy clearly improves outcomes and is considered the first-choice treatment for advanced disease, apart from the rare cases of visceral crisis.^[6]^ Adding a cyclin-dependent kinase (CDK) 4/6 inhibitor further improves outcomes.^[7]^ Premenopausal women with HR-positive metastatic breast cancer should be offered ovarian suppression or ablation plus a further endocrine agent, with or without a CDK4/6 inhibitor. We report the case of a 39-year-old woman with primary breast cancer and associated synchronous bone metastases, who experienced exceptional response achieve durable disease control, with hormonal therapy as maintenance after first line chemotherapy, with a good toxicity profile.

## 2 Case report

**Figure 1:**
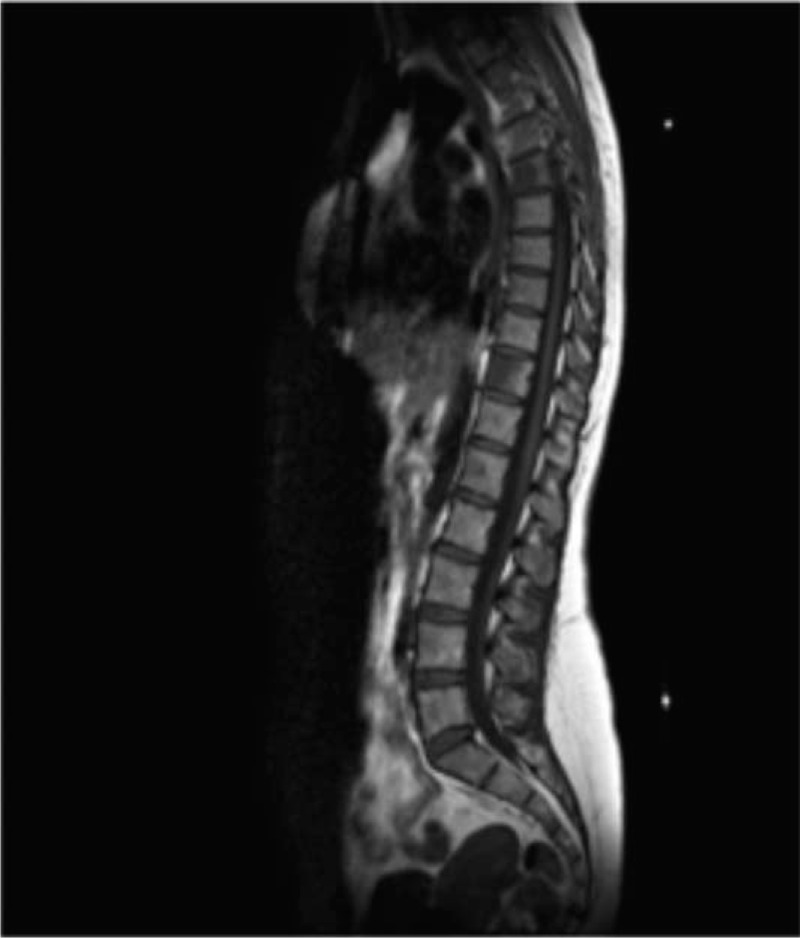
Pre-treatment MR-imaging (August 2006). MR = magnetic resonance.

**Figure 2:**
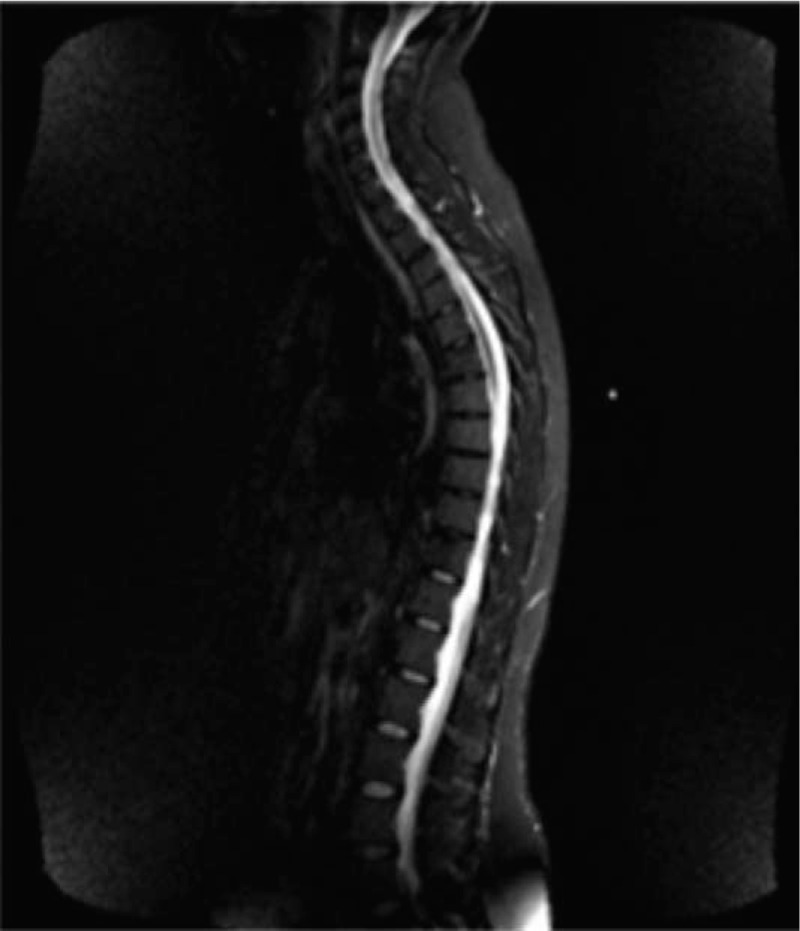
MRI after 6-mo of chemotherapy (February 2007). MRI = magnetic resonance imaging.

**Figure 3:**
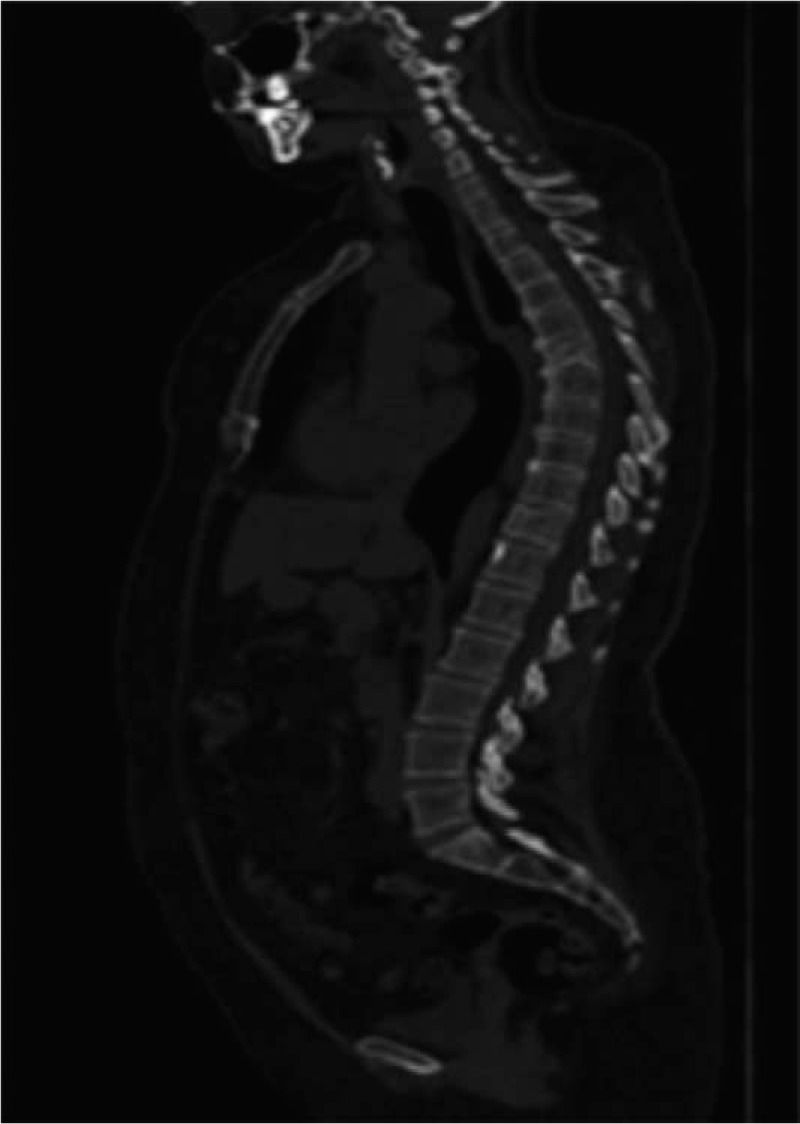
CT scan (bone window) after 12 yr of hormonal treatment for advanced breast cancer (May 2018). CT = computed tomography.

In August 2006, a 39-year-old woman underwent left mastectomy with axillary lymph node dissection. A diagnosis of moderately differentiated (G2), infiltrating carcinoma of the breast, with ductal predominant pattern, was made. The major tumor diameter was 35 mm, there was peritumoral vascular invasion, and 1 out of 33 axillary lymph nodes was metastatic. Immunohistochemistry showed that 70% of tumor cells were positive for ER and 10% for progesterone receptor (PgR), the HER2-neu score was 0 and the Ki67 labeling index was 35%. According to the pathologic stage pT2 pN1a, she performed postoperative work-up including chest X-ray and abdominal ultrasound, that resulted negative for distant metastases. A bone scan and a confirmatory magnetic resonance imaging (MRI) were also performed and showed 2 lesions at the sixth and eleventh dorsal vertebral bodies (Fig. 1). Therefore, she underwent a computed tomography (CT)-guided biopsy at the 11th dorsal vertebral body: the pathological evaluation revealed a bone metastasis of adenocarcinoma sharing immunophenotypic features with ductal carcinoma of the breast (ER 70%, PgR 0%); HER2 and Ki67 were not evaluated due to the paucity of tumor material. The definitive diagnosis was infiltrating ductal carcinoma of the breast with simultaneous bone metastases (stage IV). On August 30th, 2006, the patient had an Eastern Cooperative Oncology Group performance status of 0, the physical exam was unremarkable, and the complete blood count and blood chemistry showed no abnormal findings. The patient started a first-line chemotherapy with doxorubicin (40 mg/m^2^ intravenously [i.v.] on day 1), cyclophosphamide (500 mg/m^2^ i.v. on day 1) and weekly docetaxel (20 mg/m^2^ i.v. on days 1, 8, 15), with cycles repeated every 21 days, achieving a partial response after 3 cycles. In October 2006, after prior panoramic radiography of the jaw, zoledronic acid 4 mg i.v. every 4 weeks was added to chemotherapy. A CT scan performed after 6 cycles of chemotherapy confirmed a partial response of the disease. A bone MRI showed a major reduction of the neoplastic involvement with substitution of osteoblastic for the osteolytic lesions, especially at D6 vertebral body (Fig. 2). Given the good response observed, in February 2007 chemotherapy was discontinued after 6 cycles, and the patient started hormonal therapy with tamoxifen (20 mg daily orally) and triptorelin acetate (3.75 mg every 4 weeks, intramuscularly) as maintenance treatment, continuing zoledronic acid. In November 2007, the patient needed a dental extraction due to an abscess; therefore, the zoledronic acid was discontinued. In February 2008, a bone MRI showed maintenance of the partial remission after 12 months of tamoxifen and triptorelin. CT scans or MRI were performed every 4 to 6 months as a follow up. In May 2019, after a progression-free interval of 150 months (more than 12 years) from the first evidence of advanced disease, the imaging work-up confirmed a maintained response on bone lesions (Fig. 3) and the patient remained asymptomatic. Moreover, on May 23th, 2019, Circulating tumor cell (CTC) investigation was performed; no actual CTC was detected in the blood of the patient with this approach. At some point, the patient developed claustrophobia and refused to perform MRI; therefore, she now undergoes CT scans with bone window at lengthened intervals. Based on the maintained disease response reported by the patient, treatment was continued and is still ongoing (12 years). Up to now, no serious adverse event was reported during hormonal treatment.

## 3 Discussion

In 2006, when this patient was diagnosed with metastatic breast cancer, treatment guidelines were less stringent and CDK4/6 inhibitors were not available. In this young patient we preferred to start treatment with chemotherapy because of the young age, the PgR status (0% in the bone biopsy),^[8]^ the high grade of the tumor (G3) and the high proliferative index (Ki67 35% in the primary tumor), despite only the bones were involved by the presence of metastases. Given that in the metastatic subset of patients the primary goal is to maintain long disease control with good quality of life, after 6 cycles of chemotherapy and evidence of disease response we switched to a hormonal treatment. According to the literature data, that show better progression-free and overall survival in patients receiving combined therapy with Luteinizing Hormone-Releasing Hormone (LHRH) analog plus tamoxifen, respect to patients treated with tamoxifen alone or LHRH analog alone, we chose this type of treatment for our young patient.^[9,10]^ Given the bone metastatic involvement, our patient started also treatment with zoledronic acid.^[11]^ Bisphosphonates impact on the rate of skeletal events, reducing the risk of pathological fractures and the need for radiotherapy to control pain and to avoid medullary compression.^[12]^ Before starting this therapy, given that these drugs may be associated with the risk of osteonecrosis of the jaw, it is strongly advised to perform a dental examination for an oral hygiene session and an orthopantomography, even if in recent prospective studies the incidence of this complication was very low (1%–2%).^[13]^ To date, the optimal duration of bisphosphonates therapy has not been definitely established. The National and International Guidelines indicate to administer therapy for a period of at least 1 to 2 years, up to the entire duration of therapy for metastatic breast cancer, until there is a substantial decline in the patient's conditions.^[14]^ However, our patient permanently interrupted zoledronic acid due to the need to perform invasive dental procedures, given the possible onset of osteonecrosis of the jaw, in the context of an improved bone metastatic disease after approximately 12 months of therapy. Our report exemplifies a case of exceptional response with long-term survival of a patient with oligometastatic HR-positive breast cancer. The concept of metastatic cancer as a chronic disease has now been embraced by both physicians and patients. In rare cases, survival extends beyond what usually expected, raising the issue of curability of metastatic disease, as pointed out in recent reviews.^[15]^ Reports from chemotherapy studies conducted for metastatic breast cancer, such as those from the MD Anderson or from the Eastern Cooperative Oncology Group, suggest that a small fraction of patients, perhaps in the order of 1% to 2%, may achieve long-term (≥10 years) disease-free survival.^[16,17]^ This occurs more frequently in cases of oligometastatic disease, where up to 50% of the patients have been reported to achieve long-term survival after systemic therapy, sometimes combined with a local therapy.^[18,19]^ We cannot obviously establish if the chemotherapy, administered at our patient as first-line treatment, contributed in some way to the achievement of a long-term survival. The Cochrane's review comparing the effects of chemotherapy alone with endocrine therapy alone in metastatic breast cancer^[20]^ found that while initial treatment with chemotherapy rather than endocrine therapy may be associated with a higher response rate, the 2 initial treatments had a similar effect on overall survival. The impact of chemotherapy in case of oligometastatic disease, a condition closer to that of micrometastatic disease in radically operated breast cancer, cannot in principle be excluded, but there are no data supporting this hypothesis. Radiation therapy is delivered with some frequency to oligometastatic sites in patients with breast cancer. Although there are some hints of potential impact on long-term disease control, and although advanced techniques, such as conformal radiotherapy and hypofractionation, may reduce toxicity and improve the feasibility of treatment, demonstration of benefit from prospective randomized clinical trials is still lacking.^[21]^ Given the lack of symptoms and of risks of skeletal events, we did not prescribe radiotherapy to the metastatic lesions. A further question arising is if it is possible to hypothesize a treatment interruption after a long period of response to endocrine treatment for advanced breast cancer. Literature reports of treatment interruption after durable complete response can be found for trastuzumab,^[22–24]^ with some patients maintaining prolonged progression-free survival also after trastuzumab interruption, although data are quite scanty and this strategy is not endorsed by current guidelines. To our knowledge, there are no reports of successful experiences involving suspension of endocrine therapy after prolonged remission in metastatic breast cancer and continuing endocrine therapy until disease progression is the current standard of care. Intermittent treatment proved to be inferior to continuous treatment in metastatic breast cancer, and the Dutch Breast Cancer Research Group “Stop & Go study” study concluded that intermittent first-line treatment cannot be considered in patients with HER2-negative advanced breast cancer.^[25]^ Developing reliable methods to define the presence of microscopic residual disease could perhaps in the future help to identify patients suitable for treatment interruption after the achievement of clinical complete response.^[26,27]^ In our patient, CTC investigation was performed through a DEPArray-based assay as previously reported implemented with antibodies directed towards epithelial-mesenchymal transition and stemness markers.^[28,29]^ No actual CTC was detected in the blood of the patient with this approach, in accordance with the lasting response to therapy.

## 4 Conclusions

Our anecdotal experience highlights the existence of exceptional responders among patients with HR-positive metastatic breast cancer, who achieve clinical remission and durable disease control with endocrine therapy. Although benefit from the addition of palbociclib to endocrine therapy seems particularly relevant in patients with sensitivity to endocrine therapy^[30]^ (which certainly our patient has), the long-term disease control obtained in this case with endocrine therapy alone questions the need for using CDK4/6 inhibitors in all patients with HR-positive advanced breast cancer, highlighting the need to better study predictors of treatment benefit.

## Author contributions

Maltoni Roberta, Palleschi Michela conceived the study, wrote the article, and followed the patient. Cecconetto Lorenzo, Melegari Elisabetta, followed the patient. Rocca Andrea performed the critical revision of the article. Gallerani Giulia performed CTCs evaluation. Bravaccini Sara, Altini Mattia performed management of the data.
